# Improvement in visual function and quality of life following a blindness prevention surgery program in a rural area of Eastern China

**DOI:** 10.3892/etm.2013.1037

**Published:** 2013-04-02

**Authors:** JI-BO ZHOU, HUAI-JIN GUAN, DONG-QING ZHU, AI-MING SANG, HAI-YAN GU, XIAN-QUN FAN, SHENG-FANG GE

**Affiliations:** 1 Department of Ophthalmology, Shanghai Ninth People’s Hospital, Shanghai Jiaotong University, School of Medicine, Shanghai 200011;; 2 Department of Ophthalmology, Affiliated Hospital, Nantong University, Nantong, Jiangsu 226001;; 3 Xuhui District Center for Disease Prevention and Control, Shanghai 200011, P.R. China

**Keywords:** prevent blindness, quality of life, visual function, cataract, surgery

## Abstract

The aim of this study was to assess the changes in visual function (VF) and quality of life (QOL) among patients following blindness prevention surgery in a rural area of Eastern China. The prospective study selected cataract patients via mobile eye screening camps. VF and QOL questionnaires originally developed by Fletcher *et al* were completed prior to and 6 months after surgery. Small-incision cataract surgery (SICS) with posterior chamber intraocular lens (IOL) implantation was performed on patients by a blindness prevention surgery group. The VF and QOL scores of 178 cataract patients preoperatively were 48.58±31.18 and 65.97±26.77, respectively. The scores decreased in proportion to decreasing vision status. The VF and QOL scale scores were significantly correlated with the vision grade of the patient (r_VF_=−17.2093, t=−10.87, P<0.001, r_QOL_=−13.1399, t=−8.87, P<0.001) and age (r_VF_=−0.6505, t=−3.87, P<0.001, r_QOL_=− 0.3309, t=−2.10, P=0.037). A total of 131 patients responded to the second survey, VF and QOL scores increased significantly over a six-month postoperative period (VF=83.21±16.40, P<0.001; QOL=86.53±16.33, P<0.001). The VF scale scores were correlated with the grade of vision and residence area, the QOL scale scores were correlated with the grade of vision and gender. The VF and QOL of patients were significantly improved by performing SICS with posterior chamber IOL implantation collectively in a short period in rural areas of Eastern China. It is important to follow-up cataract patients postoperatively as untreated complications of the surgery may affect the stability of VF and QOL postoperatively.

## Introduction

Cataract is the main cause of avoidable blindness worldwide and accounts for approximately half of all cases of blindness in China (1–4). It is necessary to evaluate post-surgical outcomes not only on the basis of visual acuity, but also on self-reported visual function (VF) and vision-related quality of life (QOL). These assessments may elucidate the full spectrum of disabilities associated with visual impairment from cataracts and improve the treatment approach and rehabilitation of patients.

Several previous studies evaluated the status of cataract patients, based on the population studied in rural areas of Northern and Southern China (5–7). The cataract surgery in those studies included intracapsular cataract extraction with aphakic glasses (ICCE-AG) and extracapsular cataract extraction with posterior chamber intraocular lens (ECCE/PC-IOL). Zhao *et al* observed that the mean QOL and VF scores for the patients who underwent surgery were 61.9±30.0 and 71.0±31.8, respectively, which were similar to those in patients who had not undergone surgery and had moderate bilateral blindness (<6/60 to ≥3/60 in the better eye). The VF and QOL scores closely correlate with the visual acuity of the two patient populations (for the ICCE-AG group, r=0.64 and r=0.61, respectively, and for the ECCE/PC-IOL group, r=0.68 and r=0.59, respectively) (5). He *et al* also revealed that mean VF and QOL scores for the cataract surgery population were 41.6 and 54.5, respectively (6). These studies generally agree that cataract surgeries do not consistently produce favourable VF and QOL outcomes (7). The improvement of outcome should be given greater emphasis (5) and certain remedial efforts are required to improve the performance of local eye surgeons (6). Fletcher *et al* observed that ICCE-AG and ECCE/PC-IOL were associated with substantial benefits in improved daily VF and QOL. The patients who received ECCE/PC-IOL reported greater benefits and fewer problems with vision than the patients who received ICCE-AG (9).

Small incision cataract surgery (SICS) was recently introduced into China and performed widely in a series of rural blindness prevention activities conducted by *Sight First China Action*, while phacoemulsification is used widely in other developed areas and countries. Yao *et al* reported that microincision cataract surgery generated significantly less corneal astigmatism and improved the optical quality of the cornea by modulation transfer function (MTF) evaluation, compared with SICS. However, microincision cataract surgery showed no significant advantage in reducing corneal high order aberrations compared with SICS (10). Lam *et al* indicated that the skill transfer in their setting had superior outcomes, compared with the majority of studies in rural Asia and to the patients in their cohort treated in other facilities (11). Congdon *et al* suggested that VF was superior in their cohort and that the potential benefits of avoiding refraction and second-eye surgery were substantial. However, the acceptance of such services was modest (12). Taylor *et al* concluded that cataract surgery in Pakistan had not led to VF and QOL scores equivalent to those in unoperated individuals with the same levels of visual acuity. The higher proportion of intraocular lens (IOL) surgery in previous years was likely to improve QOL following cataract surgery. Further focus was required on rural and illiterate populations, to ensure that patients achieve comparable VF and QOL outcomes following surgery (13). However, it is unclear how much the cataract patients have benefited from the SICS with IOL implantation in the rural area of China and whether changes in VF and QOL outcomes occurred following the blindness prevention activities of *Sight First China Action,* funded by the International Lions Club. Therefore, the present study was conducted. Similar protocols have been employed previously (5–7). Chan *et al* reported that the QOL and surgical outcomes in cataract patients may be assessed by a simple questionnaire, but that the local culture and environment should be taken into consideration in the questionnaire design (14). Dam *et al* also demonstrated that a Chinese-language version of the visual function assessment (VFA) questionnaire was reliable and valid. In industrialized countries with large Chinese-speaking populations and newly developed countries of East and Southeast Asia, the VF assessment may be helpful in assisting routine clinical patient evaluation and cross-cultural outcome assessment programs. These results also suggested that self-administered VF assessments may be more reliable and valid than interview-generated assessments (15). Therefore, we consider that our results may be directly compared with the results from other population-based studies in China.

## Materials and methods

### Study design and sampling

The study was conducted in Jiangyan county, a district approximately 200 km northwest of Shanghai, in the relatively developed Jiangsu province with a primarily rural population of middle socioeconomic status. Jiangyan county is considered a good representation of geographical character, economic status, developing culture and health care conditions in the rural areas of Eastern China (16). The ethics committee of the Affiliated Hospital of Nantong University (Nantong, China) approved the survey and consent forms were obtained from each patient participating in this study.

The patients completed the survey prior to and 6 months after surgery. The patients were selected by two procedures. First, the mobile eye disease screening camps and hospitals selected the 38 villages as working sites in Jiangyan county, according to the population size and location, ensuring that there were no less than two working sites in every town of this county. The mobile eye disease screening camps were organized by the local government. The government informed the villagers of the purpose, methods and arrangement of eye examination by a local broadcasting network repeatedly, and asked the village head to be responsible for the patient organization according to the program schedule.

In these camps, local ophthalmologists and specially trained local health workers were responsible for the eye examinations, which were conducted by a flashlight and included an examination of the lids, conjunctiva, cornea, anterior chamber, iris, pupil, lens and ocular movement, without examination of the dilated fundus. A total of 2,431 patients received eye examination and 251 patients with visual acuity of the naked eye less than 6/18 in at least one eye were suspected cataract patients. The potentially eligible patients were transported to a hospital by a special bus free of charge, where they were re-examined by the members of the *Sight First China Action* cataract surgery team. A dilated fundus examination was performed on each patient. Finally, 178 patients whose corrected vision at best was <6/18 in at least one eye were identified as candidates for cataract surgery and accepted the SICS and questionnaire of VF and QOL. The patients completed the first survey and arrangements were made for the patients to be treated at two hospitals within one week, Jiangyan People’s Hospital and Jiangyan Chinese Medical Hospital (Taizhou, China). The 178 patients were instructed by the surgery doctors to travel to the surgical hospital six months later to receive free eye examinations and fill out the survey again. Of these, 131 patients accepted and 8 patients were deceased within six months.

The interviews were administered in separate rooms by trained interviewers. The patients were told that they could refuse the interview and were asked to be honest in answering the questions on the survey. Patients were informed that the personal answers would be confidential and would not be reported individually and that the answers would not affect the treatment or the cost. A few patients with slight hearing impairments finished the questionnaire with the assistance of a family member.

### VF and QOL questionnaires

The VF and QOL questionnaires used in this survey were adapted from a large-scale clinical trial of cataract surgery at the Aravind Eye Hospital (Madurai, Tamil Nadu) in India (8,9). The two questionnaires have been successfully used in surveys of blindness and cataract outcomes in Nepal (17) and in the Shunyi (5), Doumen (6) and Shatin districts (7) of China. A translation of the original English version into Chinese was modified to include some of the local dialect.

The VF questionnaire consisted of 13 items in the following subscales: general (a single question that assessed overall VF), visual perception (four questions dealing with activity limitation, near vision, intermediate vision and distance vision), sensory adaptation (six questions dealing with light/dark adaptation, visual search, color discrimination and glare disability), peripheral vision (one question) and depth perception (one question). The 12 questions in the QOL questionnaire addressed limitations of daily living due to vision problems, and contained the following subscales: self-care (including bathing, eating, dressing and toileting), mobility (including walking to neighboring houses, walking to shops and doing household chores), social (such as attending social functions and meeting friends) and mental (including feelings of being a burden on others, dejection and loss of confidence).

Using a four-point scale, each question asked about the extent to which the individual was currently experiencing a difficulty, from ‘not at all’ to ‘a lot’. The subscale scores were linearly transformed such that the response range was between 0 and 100. The composite scores for VF (VF total) and QOL (QOL total) questionnaires were calculated by equal weighting of subscale scores; weights were 1.0 for exact agreement and 0.66, 0.33 and 0 for disagreement of 1, 2 and 3 points, respectively (7).

The interviewers were trained in general interviewing techniques and administration of the questionnaire. Practice interviews were conducted on outpatients at Jiangyan People’s Hospital and Jiangyan Chinese Medical Hospital. None of the outpatients were study subjects. Intra-observer and inter-observer agreement of VF and QOL questionnaire responses were evaluated in twenty-six of the pilot studies in Jiangyan. Repeat interviews were conducted 45 min after the initial interview. A weighted kappa statistic was used to assess the degree of agreement across the original four-point rating scale of each VF and QOL item. The average kappas for intra-observer agreement (n=12) were 0.81 (range, 0.58–1.00) for VF items and 0.83 (range, 0.66–1.00) for QOL items, respectively. The average kappas for inter-observer agreement (n=14) were 0.71 (range, 0.53–0.86) for VF items and 0.82 (range, 0.57–1.0) for QOL items, respectively.

### Data analysis

Visual acuity was recorded according to normal living vision, i.e., to record the corrected visual acuity if the patient usually wears glasses, or to record the uncorrected visual acuity if the patient did not wear glasses, even if their visual acuity could be corrected with glasses.

The bilateral vision was defined by the following criteria: i) visual acuity in the better eye vs. worse eye: normal or near normal, both eyes ≥6/18 or higher; ii) visual impairment, ≥6/60 vs. <6/18 to ≥6/60; iii) unilateral blindness, ≥6/60 vs. <6/60; iv) moderate blindness, <6/60 to ≥3/60 vs. <6/60; and v) severe blindness, both eyes <3/60 (17).

SPSS 12.0 (SPSS, Inc., Chicago, IL, USA) and Epidata 2.0 (The EpiData Association, Odense, Denmark) were used to analyze the data. The correlation between vision status and VF and QOL scores was assessed by the Spearman correlation coefficients. The transformed VF and QOL scores passed a normality test. The paired Student’s t-test was used to compare the preoperative and postoperative VF and QOL scores. Multiple linear regression analysis was used to analyze the VF and QOL scores using vision status and demographic variables, and the multivariate model included the age, gender, grade of vision, residence area, disability, education, systemic disease history and previous history of ophthalmopathy. P<0.05 was considered to indicate a statistically significant result.

## Results

### Preoperative characteristics of patients

A total of 178 patients completed the survey and their average age was 69.9±9.9 years (ranging from 37 to 95 years). The majority (92.03%) of the patients lived in the country, with the remainder living in small towns. Of the patients, 60.2% were female and had an average age of 69.54 years. The average age of male patients was 70.36 years. There was no statistical difference between the average age of the males and females (P=0.522). None of the patients refused the questionnaire and none of those questioned were suffering from Alzheimer’s disease. The VF and QOL scores of 178 preoperative cases are shown in Tables I and II. It was noted by multivariate analysis of variance that the VF and QOL scores were significantly different for the various vision grades (f_VF_=10.145, P=0.000; f_QOL_=9.057, P=0.000), however, the score of visual perception in the unilateral blindness group was higher than that of the vision impairment group, and the QOL score in the unilateral blindness group was similar to that of the vision impairment group.

**Figure 1:**
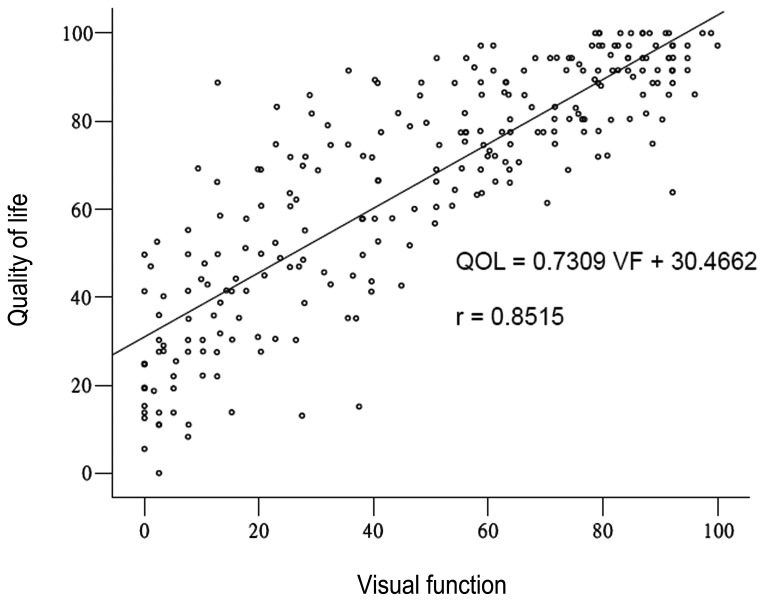
Linear regression equation of the preoperative VF and QOL scores of 178 patients. VF, visual function; QOL, quality of life.

It was revealed by multiple linear regression that the VF and QOL scores were correlated with the vision grade (r_VF_=−17.2093, t=−10.87, P=0.000, r_QOL_=−13.1399, t=−8.87, P=0.000) and age (r_VF_=−0.6505, t=−3.87, P=0.000, r_QOL_=−0.3309, t=−2.10, P=0.037) of the patient, and the far visual acuity was able to explain 37.2% of the VF results and 31.4% of the QOL results. The gender, disability, education, systemic disease history, residence area and previous history of ophthalmopathy had no statistical significance (P>0.05). The correlation coefficient for patient VF and QOL scores was statistically significant (t=25.62, P<0.001; Fig. 1).

### Postoperative patient outcomes

**Figure 2:**
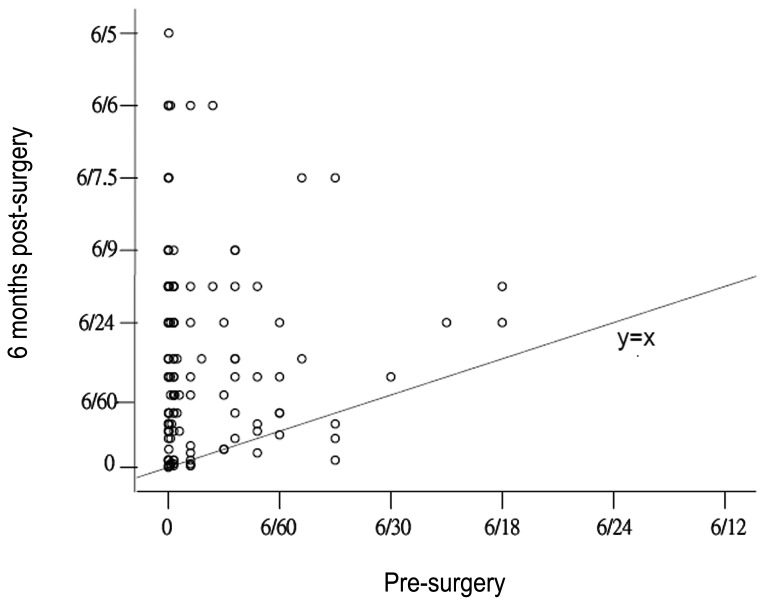
Visual acuity of the operated eye in pre- and postoperative patients. The dots above the line y=x indicate that the visual acuity improved following surgery. The dots below the line y=x indicate that the visual acuity was not improved following surgery.

Of the 178 eligible subjects, 131 were interviewed postoperatively, and 8 were deceased. The results of the visual acuity of the operated eye preoperatively and postoperatively are presented in Fig. 2. The 131 patients who responded to the postoperative survey had an average age of 69.4±9.9 years (ranging from 39 to 96 years). A total of 84 patients were female. Excluding the 8 patients who died, 77.1% of the patients completed the second survey. The preoperative data of the 131 patients was compared with the total data for 178 patients. The comparison showed that there were no significant differences in age, gender, vision grade, systemic diseases history, other disability, level of education, residence area and previous history of the eye.

**Figure 3:**
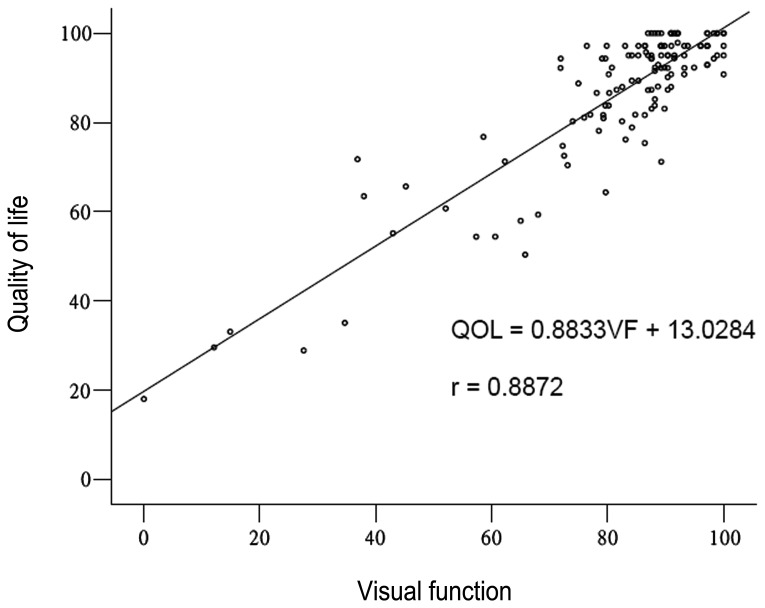
Linear regression equation of the postoperative VF and QOL scores of 131 patients. VF, visual function; QOL, quality of life.

Tables III and IV show the postoperative VF and QOL scores of the 131 patients, respectively. It was demonstrated by multiple linear regression that the VF scores were primarily correlated with the grade of vision (r=−8.3542, t=−7.51, P<0.001) and residence area (r=−3.5913, t=−2.01, P=0.047) of the patient more so than other factors. We noted that the VF of patients who lived in towns was higher than that of the patients who lived in villages. The age, gender, disability, education, systemic disease history and previous history of ophthalmopathy had no statistical significance (P>0.05). The outcomes also suggested that the QOL scores were mainly correlated with the grade of vision (r=−9.9501, t=−10.27, P<0.001) and gender of the patient (r=−6.7718, t=−2.59, P=0.011), since the QOL of female patients appeared to be lower than that of the males. The age, disability, education, systemic disease history, residence area and previous history of ophthalmopathy had no statistical significance (P>0.05). The correlation coefficient of VF and QOL of the patients was statistically significant (t=21.84, P<0.001; Fig. 3).

The preoperative VF and QOL scores of 131 patients were compared with those postoperatively (Table III and IV). There were statistically significant differences between the pre- and postoperative VF and QOL scores of the 131 patients (Table V). Table VI illustrates the comparison of VF and QOL scores preoperatively and postoperatively in 131 patients divided by age. The Spearman’s correlation coefficient between the VF change and age was 0.176 (P=0.044), and the Spearman’s correlation coefficient between the QOL change and age was 0.123 (P=0.162). The mean VF change significantly correlated with the mean QOL change in the 131 patients (r=0.767, P<0.001).

## Discussion

In the current study the preoperative results indicated that the mean scores of VF and QOL and their subscales were directly correlated with vision status (8). It was noteworthy that the scores of visual perception of the unilateral blindness group were higher than those of the vision impairment group (t=2.018, P=0.045), which may be explained by the better eye phenomena. This was different from the findings of previous studies (5–7). However, the mean QOL scores of the unilateral blindness group were similar to those of the vision impairment group (t=0.243, P=0.809) and there was a large difference between the moderate blindness group and the severe blindness group (t=3.102, P=0.003). As identified in the survey, the VF and QOL mean scores were significantly influenced by factors other than visual status, such as character traits and cultural background. This may partly explain why the social and mental Spearman’s correlation coefficients were low for the visual status. In the unilateral blindness group, the self-care (t=0.302, P=0.763) and social capabilities (t=0.005, P=0.996) were similar to those of the vision impairment group. As observed in other studies (5–7), the mean VF and QOL scores were not significantly correlated with age, gender or education levels. In the current study, the mean VF and QOL scores were also not correlated significantly with the previous history of ophthalmopathy. A likely explanation is that the selected patients all had surgical indications, regardless of disease severity and age, and that certain patients with severe eye diseases, such as corneal leucoma, were excluded in the preoperative screening.

The scores of preoperative VF and QOL indicated that the patients in the severe blindness group were in most need of improvement. If the visual acuity of one eye improved to 6/60, the VF and QOL scores would improve significantly (18). In our opinion, in a situation of limited resources, patients with severe blindness should be prioritized in order to make the procedure more cost-effective and efficient. In order to prevent the marked decrease of the VF and QOL of cataract patients, medical intervention should occur prior to the onset of severe blindness. We also observed that cataract patients suffered from psychological problems, as numerous patients had inferiority complexes. Limited activity and psychological inferiority resulted in a decrease in the QOL, which led to the conclusion that attention should be provided for the mental health problems of cataract patients.

We also observed that there were significant differences in the VF and QOL scores prior to and following surgery in the 131 patients. The VF and QOL outcomes improved significantly following surgery (19), particularly in the general subscale of VF and the mental subscale of QOL. The VF scores of the vision impairment group were similar to those of the unilateral blindness group and depth perception was the highest subscale of VF. The QOL scores of the normal group were almost equal to those of the vision impairment group. The greatest difference in QOL mean scores was between the moderate blindness group and the severe blindness group. The four subscales of the QOL were close. The VF mean scores of the patients who lived in towns were higher than those from patients who lived in villages, partly due to the difference in living facilities. The QOL scores of female patients were lower than those of male patients, perhaps partly due to housework. In general, the responsibilities of women in the household require better vision. Our results were similar to those obtained for Hong Kong and the VF and QOL scores were better than those observed in Shunyi and Doumen (5–7), in which the outcomes were based on the whole population. Thus, our results suggest that, performing SICS and IOL implantation collectively in a short period was effective in a rural area of China.

In our survey, 6.1% of the patients had moderate blindness and 8.4% of the patients had severe blindness. Such patients were not able to recover, mainly due to complications such as pathological myopia, glaucoma, retinal degeneration, optic nerve atrophy, vitreous opacity and posterior capsular opacities. In addition, we also identified a patient with high myopia who was inappropriately implanted with a normal IOL due to a lack of examination equipment and unknown myopic history. A routine follow-up affected the quality of the surgery. We observed that certain patients with refractive error, trichiasis, chronic conjunctivitis, uveitis, posterior capsular opacities and age-related macular degeneration required additional postoperative treatment. These factors impacted the VF and QOL outcomes of the surgery (20).

The majority of the patients surveyed in this project were those with a lower educational background, who were not able to obtain cataract surgery independently and required subsidies. Evaluating the outcomes of this surgery will comprehensively appraise the outcomes of blindness prevention projects. The survey of the 178 patients was considered as a baseline. Due to the difficulties of follow-up, the subjects were not selected randomly. Oliver *et al* observed that only 41.4% cataract patients returned for a 12-month follow-up visit and completed the VF and QOL questionnaires in South India, despite transport and a meal on the day of the follow-up visit being offered free of charge to all eligible patients (21). Minassian *et al* invited patients to a study comparing the effect of different cataract surgeries (SICS and phacoemulsification) in England. The patients who completed the trial in 1 year accounted for the 67.4% of the total patients invited to participate (22). In the present study, patients were informed that we required the true results, good or bad, and attempted to avoid the dissatisfied subjects failing to respond to the study. However, only 131 patients that were contacted responded to the second survey, potentially resulting in certain bias (23). Additionally, establishing mobile eye camps in the villages may persuade patients to accept the postoperative survey.

Measuring QOL has advantages in assessing the costs and benefits. However, the floor-and-ceiling effect should not be ignored. It is possible that negative effects of cataract are overestimated and the benefits of treatment are underestimated (9). It is important to comprehensively understand the correlation between vision status and the VF and QOL questionnaires. The VF and QOL assessments capture vision deficit and individual adaptation to vision deficit. Vision deficits affect different people differently (5). The self report reflects the personal level and the vision status reflects the eye level. We observed there were high correlations between the two questionnaires, preand postoperatively (Figs. 1 and 3, respectively). Gothwal *et al* suggested that the QOL questionnaire was not a valid measure of QOL. However, the VF questionnaire was a reliable and valid measure of visual disability in patients with cataract, and although targeting was suboptimal in a developed country, it may be optimal in a developing country as was originally intended (24). We suggest that the two questionnaires are required due to their differences in numerous aspects. The VF subscale emphasized the problems of vision function directly. The QOL subscale was designed to focus specifically on those activities that are important in daily living for which vision impairment may have an adverse impact (8). Therefore, it is as predicted that the VF score changed more dramatically than the QOL score.

In conclusion, the VF and QOL scores of cataract patients increased significantly following surgery, suggesting that performing SICS with posterior chamber IOL implantation collectively in a short period is an effective method for improving the QOL of cataract patients in rural areas, as observed in this Blindness Prevention Surgery Program in Eastern China. However, the patient follow-up is important and should be conducted carefully, as the untreated complications of the surgery may affect the stability of patient postoperative VF and QOL.

## Figures and Tables

**Table I:** Preoperative visual function (VF) scores by category of visual acuity (mean ± SD).

Variable	Vision category					Spearman correlation coefficient
	Vision impairment	Unilateral blindness	Moderate blindness	Severe blindness	All	
No. of cases	33 (25.19%)	9 (6.87%)	77 (58.78%)	12 (9.16%)	131	
General	44.08±9.46	39.92±8.63	29.33±5.80	17.00±6.75	33.80±27.71	−0.41
Visual perception	51.17±25.30	61.99±26.42	38.69±29.65	17.78±18.74	48.26±30.95	−0.57
Peripheral vision	71.10±25.29	72.33±24.63	49.76±25.74	25.77±21.43	58.94±31.10	−0.54
Sensory adaptation	60.92±42.24	61.75±41.80	25.78±36.32	8.00±18.50	45.66±43.59	−0.60
Depth perception	44.08±35.55	49.08±37.63	11.00±16.50	10.00±17.47	36.16±36.72	−0.50
Total VF	60.03±23.87	64.05±24.94	39.96±23.99	20.06±16.86	50.95±29.62	−0.60

**Table II:** Preoperative quality of life (QOL) scores by category of visual acuity (mean ± SD).

Variable	Vision category					Spearman correlation coefficient
	Vision impairment	Unilateral blindness	Moderate blindness	Severe blindness	All	
No. of cases	33 (25.19%)	9 (6.87%)	77 (58.78%)	12 (9.16%)	131	
Self-care	92.31±20.04	86.09±20.42	68.22±29.25	44.94±31.34	75.07±30.09	−0.55
Mobility	82.19±20.00	79.88±24.19	57.78±30.86	36.48±31.51	67.64±32.25	−0.54
Social	72.13±35.10	80.42±27.19	72.06±29.00	57.42±30.08	73.29±30.08	−0.36
Mental	74.81±27.78	60.03±25.66	52.70±28.17	39.45±26.64	55.70±28.04	−0.35
Total QOL	82.04±16.41	77.08±19.01	62.37±23.93	43.54±22.93	68.07±24.90	−0.55

**Table III:** Postoperative visual function (VF) scores by category of visual acuity (mean ± SD).

Variable	Vision Category						Spearman correlation coefficient
	Normal	Vision impairment	Unilateral blindness	Moderate blindness	Severe blindness	All	
No. of cases	26 (19.8%)	40 (30.5%)	46 (35.1%)	8 (6.1%)	11 (8.4%)	131	
General	79.12±19.32	64.50±21.33	63.26±20.90	45.63±39.59	39.00±28.84	63.67±25.05	−0.37
Visual perception	91.83±7.00	87.50±11.92	85.08±11.42	62.09±14.06	43.75±34.49	82.28±19.48	−0.47
Peripheral vision	98.69±6.67	94.08±15.08	65.57±11.58	62.13±27.93	45.18±37.26	89.46±22.82	−0.44
Sensory adaptation	91.29±7.37	85.90±10.62	88.59±9.07	70.50±9.80	60.36±30.04	84.83±14.98	−0.32
Depth perception	100.00±0.00	95.78±13.63	93.37±15.32	74.75±29.74	48.18±34.48	90.49±21.73	−0.47
Total VF	91.76±5.75	86.13±10.18	86.47±8.82	65.68±12.12	51.50±30.55	83.21±16.40	−0.48

**Table IV:** Postoperative quality of life (QOL) scores by category of visual acuity (mean ± SD).

Variable	Vision category						Spearman correlation coefficient
	Normal	Vision impairment	Unilateral blindness	Moderate blindness	Severe blindness	All	
No. of cases	26 (19.8%)	40 (30.5%)	46 (35.1%)	8 (6.1%)	11 (8.4%)	131	
Self-care	98.37±4.18	94.26±9.11	88.19±11.50	73.50±13.07	52.70±29.80	88.18±17.41	−0.65
Mobility	92.62±7.76	87.60±7.94	80.93±12.87	67.79±11.03	44.15±22.72	81.40±17.31	−0.61
Social	99.35±3.33	96.61±8.73	90.08±14.87	66.44±21.88	55.82±29.14	89.59±18.88	−0.58
Mental	98.26±4.17	91.86±15.21	87.53±16.43	71.88±18.01	56.27±31.21	87.40±19.63	−0.48
Total QOL	97.06±3.87	92.39±8.33	86.53±11.06	70.49±11.49	51.98±23.91	86.53±16.33	−0.68

**Table V:** Pre- and postoperative comparison of VF and QOL in 131 patients (Student’s t-test).

Variable	t	P-value
Total VF	−11.05	<0.0001
General	−9.15	<0.0001
Visual perception	−10.65	<0.0001
Peripheral vision	−9.33	<0.0001
Sensory adaptation	−9.98	<0.0001
Depth perception	−8.08	<0.0001
Total QOL	−7.30	<0.0001
Self-care	−4.32	0.0159
Mobility	−4.30	0.0090
Social	−5.25	<0.0001
Mental	−10.60	<0.0001

VF, visual function; QOL, quality of life.

**Table VI:** Pre- and postoperative comparison of QOL and VF between 131 patients divided by patient age (Student’s t-test).

Age (years)	Case (%)	VF				QOL			
	
		Pr	Po	t	P-value	Pr	Po	t	P-value
<50	6 (4.58)	50.67±35.02	75.66±24.55	2.433	0.059	65.85±30.64	82.17±27.67	2.208	0.078
50–59	22 (16.79)	53.59±30.58	81.71±12.66	5.062	<0.0001	71.01±22.42	84.06±15.60	3.749	<0.0001
60–69	43 (32.82)	59.23±25.89	88.01±7.89	7.246	<0.0001	74.86±21.23	91.46±8.58	5.691	<0.00001
70–79	52 (39.69)	40.15±27.37	77.41±23.65	9.179	<0.0001	61.37±25.63	83.58±19.99	6.033	<0.0001
≥80	8 (6.11)	44.18±31.35	87.53±10.20	4.589	0.003	66.36±26.79	91.11±8.81	3.256	0.014
Total	131 (100)	49.40±28.86	82.15±17.84	13.658	<0.0001	67.92±24.38	86.64±16.38	9.681	<0.0001

Pr, preoperative; Po, postoperative; VF, visual function; QOL, quality of life.
